# Organic electrochromic energy storage materials and device design

**DOI:** 10.3389/fchem.2022.1001425

**Published:** 2022-09-23

**Authors:** Qingjiang Liu, Liangliang Yang, Wei Ling, Binbin Guo, Lina Chen, Jiaqi Wang, Jiaolong Zhang, Wenhui Wang, Funian Mo

**Affiliations:** ^1^ Sauvage Laboratory for Smart Materials, School of Materials Science and Engineering, Harbin Institute of Technology, Shenzhen, China; ^2^ School of Mechatronics Engineering, Harbin Institute of Technology, Harbin, China; ^3^ School of Materials Science and Engineering, Dongguan University of Technology, Dongguan, China; ^4^ Department of Civil and Environmental Engineering, Harbin Institute of Technology, Shenzhen, China

**Keywords:** organic materials, polymer, electrochromic, energy storage, multifunction

## Abstract

While not affecting electrochemical performance of energy storage devices, integrating multi-functional properties such as electrochromic functions into energy storage devices can effectively promote the development of multifunctional devices. Compared with inorganic electrochromic materials, organic materials possess the significant advantages of facile preparation, low cost, and large color contrast. Specifically, most polymer materials show excellent electrochemical properties, which can be widely used in the design and development of energy storage devices. In this article, we focus on the application of organic electrochromic materials in energy storage devices. The working mechanisms, electrochemical performance of different types of organics as well as the shortcomings of organic electrochromic materials in related devices are discussed in detail.

## Introduction

Electrochromism refers to the phenomenon of REDOX reaction accompanied by color change or transmittance change, when the material is changed by external voltage or current (Davy et al., 2017; Zhang et al., 2019a; Cai et al., 2020a; Jang et al., 2021). It is very similar to the energy conversion process of energy storage devices, so more and more people are applying electrochromic materials in the field of multifunctional energy storage, which can not only achieve excellent electrochemical performance, but also monitor the status of energy storage devices (Yang et al., 2019; Zhai et al., 2019; Dewan et al., 2022; Wang et al., 2022). There are many functional materials that can achieve electrochromism, such as WO_3_, NiO, TiO_2_, V_2_O_5_ and other metal oxides (Zhang et al., 2019b; Kim et al., 2020a; Lee et al., 2020; Shi et al., 2020; Zhang et al., 2020; Zhou et al., 2020). However, most inorganic materials are faced with problems including poor conductivity, low color conversion sensitivity, low color contrast and poor electrochemical performance when applied in energy storage devices (Yun et al., 2017; Li et al., 2019a; Liu et al., 2019; Liu et al., 2020a; Chen et al., 2020; Guo et al., 2021; Lei et al., 2021; Poh et al., 2021; Cai et al., 2022). Moreover, electrochromic color changes of inorganic materials are relatively simplex (Elool Dov et al., 2017; Cai et al., 2020b), and it is difficult to realize the advantages of high capacity, good cycling stability and high energy density of energy storage devices (Laschuk et al., 2020; Li et al., 2021). In contrast, most of the polymer materials show excellent electrochemical performance (Guo et al., 2017; Poh et al., 2021), and the color contrast is large after electrochromic, so the materials used in organic discoloration (Li et al., 2019b; Wang et al., 2021), have gained much attention in energy storage field because it can not only establish intelligent energy storage device (Cai et al., 2016; Li et al., 2020), but also promote the use of consumer experience and the development of artificial intelligence equipment and progress (Sassi et al., 2016; An et al., 2018). In electrochromic energy storage devices, the color changes of materials need to be clearly observed all the time (Kim et al., 2018; Kim et al., 2020b; In et al., 2020). Therefore, their packaging method is different from traditional energy storage devices (Huang et al., 2018; Liu et al., 2020b; Pei et al., 2020). Electrochromic devices generally adopt multi-layer structure including double electrode layer, electrolyte layer and collector layer, and the typical collector layer is transparent indium tin oxide (ITO) conductive glass (Huang et al., 2016; Zhang et al., 2017; Qin et al., 2018; Li et al., 2019c). When constructing multifunctional energy storage devices, it is necessary to select appropriate electrode materials and ensure the materials can maintain good energy conversion and electrochromic reversibility and stability (Salles et al., 2019; Jia et al., 2021). Hence, we have to consider the influence of electrolyte on the performance of electrochromic materials when ions are removed from or released into electrolyte.

In this article, we first briefly summarize the types of organic electrochromic materials, the basic working mechanism and applications in various fields of energy storage including batteries, supercapacitors and solar cells. Secondly, electrochemical and electrochromic properties of organic electrochromic materials in different energy storage devices are summarized and analyzed, in order to obtain multifunctional energy storage devices with both excellent electrochemical energy conversion performance and stable electrochromic properties, so as to promote the development of organic electrochromic materials in energy storage. Finally, constructive viewpoints are put forward in order to promote the mass production application of organic electrochromic materials in the field of energy storage.

## Electrochromic materials and mechanisms

**FIGURE 1:**
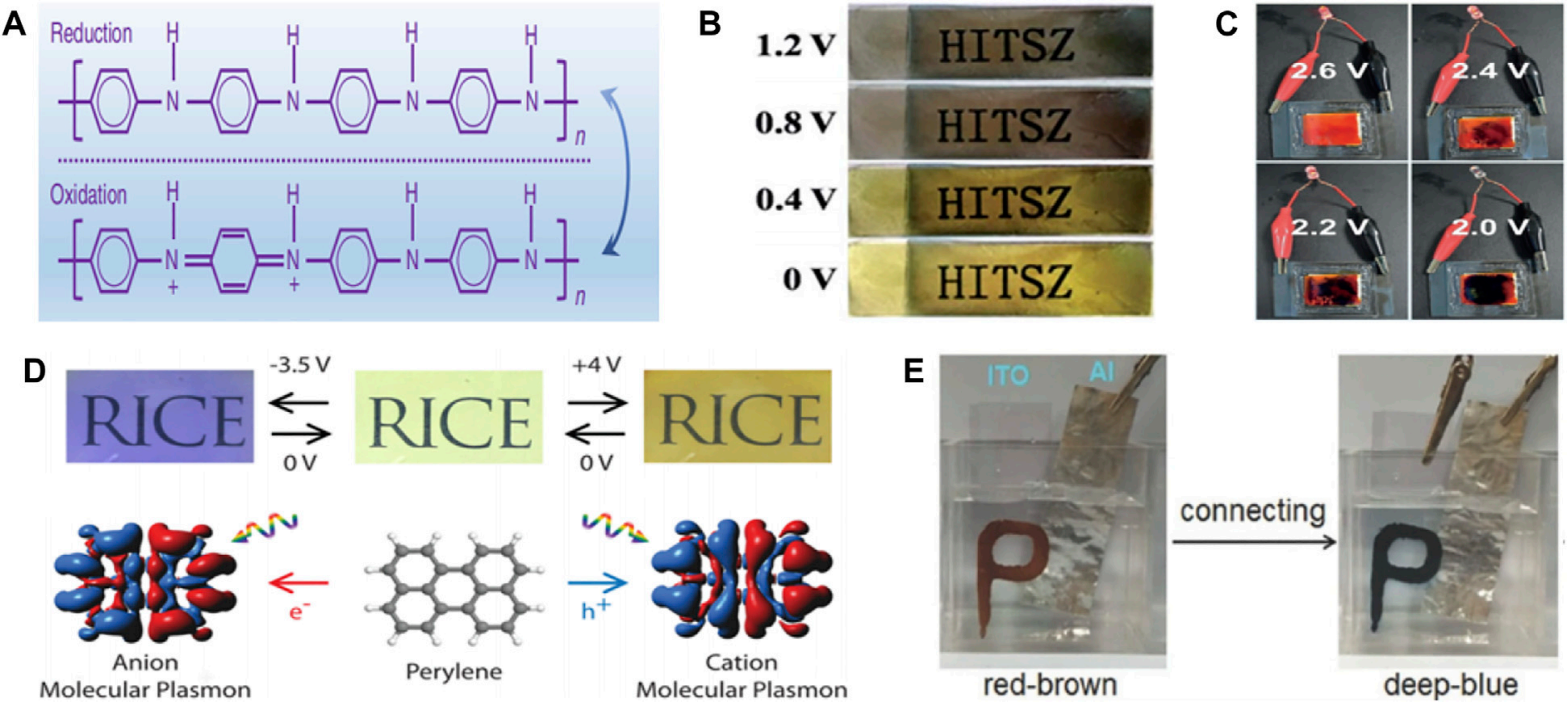
**(A)** chemical structures of reduced and oxidized forms of PANI. (Xu et al., 2016) with permission from Springer. **(B)** Photos of PPy at different voltages. (Wang et al., 2018) with permission from Royal Society of Chemistry. **(C)** Color change of poly (chalcogenoviologen)s when discharged from 2.6 to 2.0 V. (Li et al., 2019d) with permission from Wiley-VCH. **(D)** The PAHs-based material enables reversible switching of multiple colors when applied voltages of -3.5, 0, and +4 V. (Stec et al., 2017) with permission from American Chemical Society. **(E)** Photo of NA/H_6_P_2_W_18_O_62_-based device electrochromic. (Li et al., 2018a) with permission from Wiley-VCH.

Polyaniline (PANI) is one of the most commonly used organic electrochromic material (Tong et al., 2022). Different from general inorganic materials, PANI has a stabilizing effect on electrically induced discoloration and shows excellent electrochemical performance simultaneously (Zhang et al., 2018). PANI has been widely used in electrode materials of batteries and supercapacitors due to its facile synthesis and low cost (Tong et al., 2021). As shown in Figure 1A, when the voltage is applied, the REDOX reaction of PANI is induced, and the material changes gradually from yellow reducing state to green oxidation state (Xu et al., 2016). It may show a distinct color differentiation from blue or black when electrolyte is changed. Similarly, polypyrrole (PPy) is also widely used in the field of organic electrochromic energy storage materials. When the charging voltage reaches 1.2 V, PPy will show a black state. With the decrease of voltage and capacity, the black area continuously decreases and the yellow area gradually increases. The material completely changes to the yellow state at 0 V. It is worth noting that the black state could be reversibly recovered when the voltage is recharged to 1.2 V (Figure 1B). During charging and discharging, ions in the electrolyte will be inserted into and released from PPy, leading to REDOX reaction of the material (Wang et al., 2018). In addition, PPy also shows self-charging performance, which can use O_2_ in air to return to black oxidation state and restore the specific capacity of the device (Yang et al., 2019). Some conjugated polymers such as polymer poly (4,7- bis(5-(2,3-dihydrothieno [3,4-b] [1,4] dioxin-5-yl)-3,4-bis(hexyloxy)thiophen-2-yl) benzo [c] [1,2,5] thiadiazole) (poly (BT-Th-EDOT)) can also achieve electrochromism. These polymers are oxidized and their colour changes from green to blue when the voltage is changed (Ming et al., 2020). This is due to *π*-π* transitions in conjugated blocks and charge transfer between donor and acceptor units. Moreover, when the voltage increases from -0.2 to 0.2 V, the original double absorption peak becomes weaker and a new absorption peak is formed, which corresponds to the emergence of new conjugated polymer polaron. Due to the polaron to bipolaron transformation, the intensity of the emerging absorption peak decreases until the polymer completely turns blue at 1 V.

Similarly, viologens (1,1′-Disubstituted-4,4′-bipyridinium salt) is also a common polymer in the field of electrochromism. When the applied current or voltage changes, a two-step reduction reaction (RV^2+^ + e^−^↔ RV^+^, RV^+^ + e^−^↔RV) occurs, accompanied by obvious color change. However, when it is applied to electrochemical energy storage devices, it is difficult to show satisfactory electrochemical and electrochromic performance. However, its properties can be effectively improved by doping with other elements. As shown in Figure 1C, poly (chalcogenoviologen)s is prepared by copolymerization of sulfur element atoms with violet based polymer. It starts to discharge from 2.6 V, and gradually changes from bright red to dark purple with the continuous decrease of voltage, which suggests that electrochromic materials can act as intelligent monitoring of the state of storage device (Li et al., 2019d). Polycyclic aromatic hydrocarbons (PAHs) are also considered as organic electrochromism materials, which can change from colorless state to colored state when charged. For example, the electrochromic devices made by Naomi et al. based on PAHs possess the characteristics of reversible switching between multiple colors, namely colorless (0 V), olive (+4 V) and royal blue (-3.5 V), and can reversibly transform for more than 100 times (Figure 1D). The reversible color switching is attributed to the movement of electrons or holes under the applied potential, which excites plasma plasmons and changes the material properties accordingly. When the applied potential is removed, the PAHs return to colorless state with excellent reversibility (Stec et al., 2017). There are also some polymers that can undergo multi-electron REDOX reactions when the applied voltage changes, accompanied by visual color changes, such as hexaza trinaphthalene polymers. When the voltage is applied, the cation in the electrolyte will be adsorbed by the N and N lone electron pairs in the polymer, forming new chemical bonds, thereby resulting in the phenomenon of red shift in the absorption spectrum of the polymer. A reverse and reversible process occurs during discharging, which leads to the recovery of the material to its original properties (Chen et al., 2021). The electrochromic properties of polymers can be further stabilized by combining multiple polymers to prepare new materials. For example, Li et al. (Li et al., 2018a) combined the heteropolyacid H_6_P_2_W_18_O_62_ with the water-immiscible amino acid 3-(2-naphthyl)-l-alanine (NA) to prepare a reddish-brown NA/H_6_P_2_W_18_O_62_ composite. The material was able to spontaneously switch to a dark blue color and return to its original reddish-brown color when oxidized by H_2_O_2_. This reversible process is attributed to the reduction of W^6+^ in H_6_P_2_W_18_O_62_ to W^5+^, corresponding to the color change from reddish-brown to dark blue. H_2_O_2_ has strong oxidizing property and can re-oxidize W^5+^ to W^6+^, which also indicates the excellent self-powering property of NA/H_6_P_2_W_18_O_62_ composite (Figure 1E). In addition, hydrogen-bonded organic skeletons (HOFs) can also achieve reversible electrochromic effects. Feng et al. prepared highly porous HOFs films with electrochromic multifunctional function by electrophoretic deposition, which can achieve reversible switching between yellow and blue-violet, resulting in a transition between 75 and 25% light transmittance of smart glass originated from the REDOX transformation of the pyrene part of the materials ligands (Feng et al., 2020).

## Electrochromic energy storage devices

The occurrence of electrochromic materials is accompanied by redox reactions and intercalation/deintercalation of ions, and the state of energy storage devices can be visually monitored according to the color of the material. Therefore, electrochromic materials show great potential and application prospects in energy conversion devices (Li et al., 2018b; Liang et al., 2018). Among different kinds of electrochromic materials, organic electrochromic materials are widely used as electrode materials for multifunctional energy storage devices due to their excellent characteristics of easy synthesis, low cost, stable performance, and large color contrast (Zhu et al., 2018). The Zn-based and Al-based energy storage devices can perform electrochemical energy storage conversion in air, and most of the Zn-based and Al-based electrolytes are colorless and do not cause color interference (Ji et al., 2020; Liu et al., 2022). Hence, organic electrochromic materials have attracted much attention in Zn-based and Al-based energy storage devices (Huang et al., 2015; Mo et al., 2019; Eh et al., 2021). More importantly, when electrochromic materials are applied to energy storage, their electrochromic and electrochemical performance stability will be affected. During the conversion of electrochemical energy storage, the current and the composition of electrolyte will affect the characteristics of the material itself. Therefore, we are committed to developing a multifunctional energy storage device with excellent electrochromic and electrochemical performance stability at the same time.

### Electrochromic battery

**FIGURE 2:**
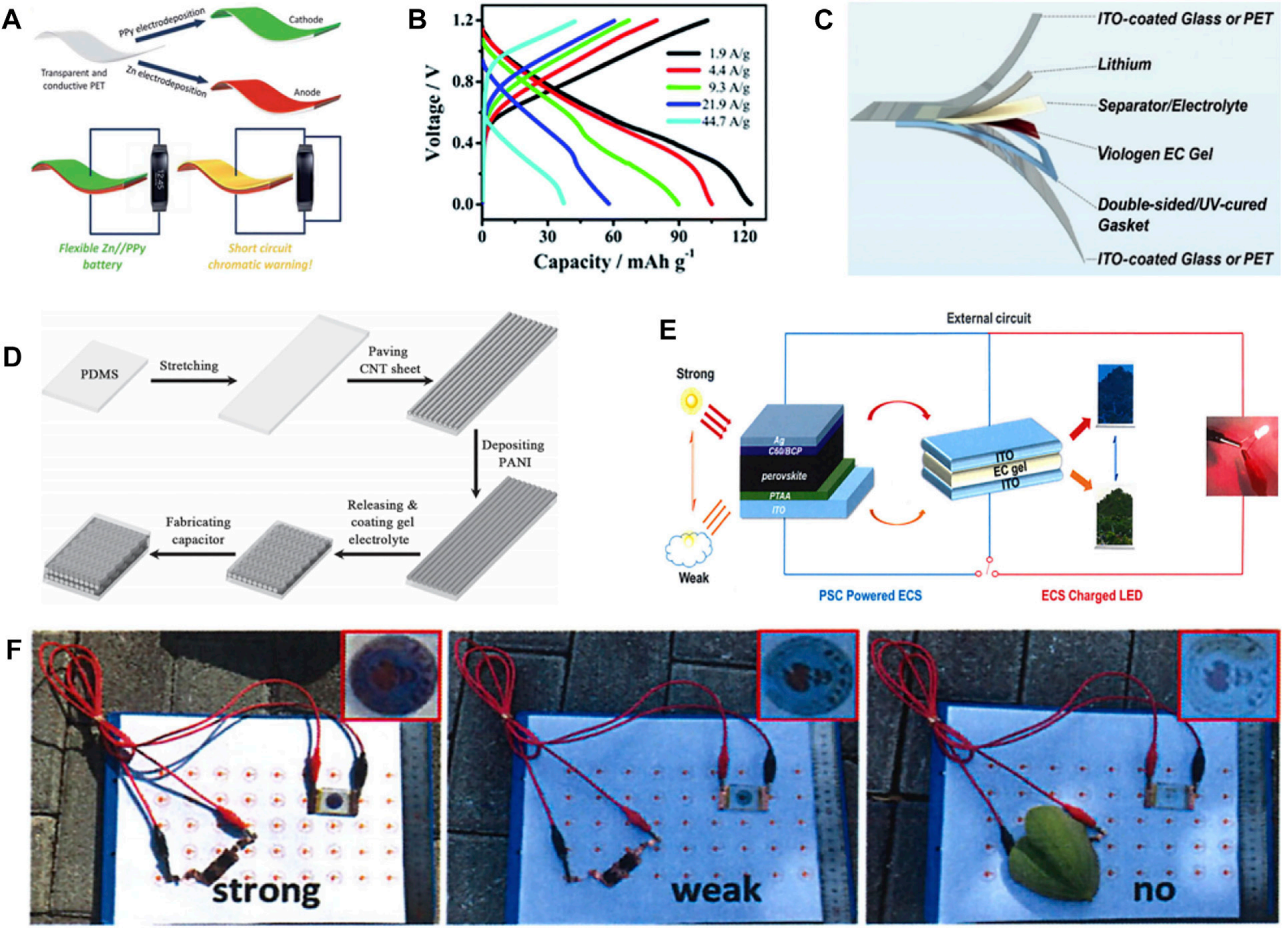
**(A)** Schematic diagram of Zn//PPy battery with short-circuit warning function. **(B)** GCD curves of Zn//PPy battery at different current densities. (Wang et al., 2018) with permission from Royal Society of Chemistry. **(C)** Flexible ECLIB based on Poly (chalcogenoviologen). (Li G. et al., 2019) with permission from Wiley-VCH. **(D)** Schematic diagram of the fabrication of stretchable ECSCs. (Chen et al., 2014) with permission from Wiley-VCH. **(E)** Schematic diagram of the electrochromic device driven by PSC. **(F)** The photos of PSC-powered electrochromic device under different light intensities. (Ling et al., 2021) with permission from Springer.

Wang et al. (Wang et al., 2018) used PPy as the cathode of electrochromic Zinc ion battery (ECZIB) to construct Zn//PPy electrochromic battery with polyvinyl alcohol-based gel electrolyte and zinc anode electrode (Figure 2A). The rechargeable battery has wearable features and short-circuit warning capabilities. When the voltage of the wearable energy storage device goes to be 0 V in the process of wearing, that is, in the short-circuit state, the PPy electrode can respond quickly and immediately by turning yellow to provide visual energy storage information. Moreover, the battery persists to show excellent electrochemical performance in different bending states, and can stably power the device. The transparent state of the device will not be affected in the process of electrochromism. ECZIB delivers a high capacity of 123 mAh g^−1^ at the current density of 1.9 A g^−1^, and has rapid charging characteristics (Figure 2B). Thanks to its excellent electrochromic performance and stable electrochemical performance, polyaniline has been studied and modified by more and more people, and great progress has been made. For example, Wang et al. (Wang et al., 2020) used aniline and aniline-2, 5-disulfonic acid co-polymerization to prepare self-doped polyaniline electrode. The self-doped polyaniline electrode material has superior electrochemical performance than PANI. It exhibits a specific capacity of 180.5 mAh g^−1^ at a current density of 0.5 A g^−1^ as well as good rate performance. Even when the current density is increased to 10 A g^−1^, it still has 136 mAh g^−1^, which is 75.3% of the capacity obtained at 0.5 A g^−1^. Moreover, the capacity retention is as high as 80% after 1,000 cycles at 5 A g^−1^, indicating superior stability. The battery assembled with self-doped polyaniline electrode owns remarkable energy storage condition monitoring performance. It shows obvious color transformation between light yellow, green and dark green in the voltage range of 0.51.6 V. Yellow color manifests the poor state of ECZIB, while dark green indicates full charge state. These studies could promote the development of multifunctional energy storage devices.

In addition to zinc ion batteries, electrochromic aluminum ion batteries (ECAIB) also show great potential in the field of multifunctional energy storage. However, when the REDOX reaction occurs on the surface of Al, a passivation layer is easily formed, which will hinder the subsequent chemical reaction. Therefore, the cycle stability of aluminum ion battery is very poor (Sun et al., 2020a). Lv et al. (2021) assembled stream ECAIB using high concentration organic aluminum salt (5 MAl(TOF)3 and 1 M H3PO4) as mixed electrolyte, PANI as cathode and aluminum as anode, which shows effective inhibition effect on the formation of aluminum passivation. This phenomenon is attributed to the formation of complex ions between Al^3+^, H_2_PO_4_^−^, TOF^−^ in the electrolyte, which accelerates the reaction kinetics and improves the cyclic stability and rate performance of ECAIB. The ECAIB still gives a specific capacity of 51 mAh g^−1^ after 3,850 cycles at 2 A g^−1^, corresponding to a capacity retention of 58%. Moreover, the ECAIB has excellent rate performance, with specific capacity of 167 mAh g^−1^ and 61 mAh g^−1^ at 0.5 A g^−1^ and 2.5 A g^−1^ respectively, and with specific capacity of 225 mAh g^−1^ when the current is restored to 0.5 A g^−1^ again due to the activation process of the material. Notably, the ECAIB exhibits a high coloring efficiency of 84 cm^2^ C^−1^ at 630 nm and has an open circuit voltage of 1.2 V in the coloring state, which can power an electronic clock. It will change to transparent state when discharging to 0.6 V or short circuit, reflecting the great potential and application prospect of multi-functional energy storage devices.

In addition to polyvalent ion batteries, monovalent lithium ion battery electrode materials can also achieve electrochromism. Li et al. (2019d) constructed electrochromic lithium ion battery (ECLIB) using poly (chalcogenoviologen)s doped with S, Se and other elements as anode (Figure 2C). ECLIB shows a specific capacity of 799 mAh g^−1^ at 0.05 A g^−1^, and it transforms from red to purple during discharge. Hence, it also owns the function of monitoring the state of battery storage. Chen et al. (2021) used hexaazatrinaphthylene-based polymer as cathode for ECLIB. It exhibits stable electrochromic properties during charge and discharge, with the ability to switch from orange to pink and then to green. Surface area of the cathode material is increased due to the polymer’s multi-pore structure, which not only achieves a high voltage discharge platform of 3.75 V, but also enables a high discharge specific capacity of 168 mAh g^−1^ and stable rate performance. It is noteworthy that the traditional electrochromic inorganic and organic materials can be copolymerized into composite materials, which can achieve stable electrochromic performance and excellent electrochemical properties concurrently. For example, Zhang et al. (2018) used the composite material composed of WO_3_ and PANI as cathode for ECLIB. WO_3_ and PANI could realize color complementarity during charging and discharging, thus achieving diversified color changes (reversible switching between purple, green, yellow, gray and blue) and stable cyclic discoloration over 1,200 times. The composite electrode material also has a fast response speed and can switch between coloring and bleaching in less than 2 s.

### Electrochromic supercapacitors

Compared with battery devices, supercapacitors possess (Chen et al., 2014; Cai et al., 2015) the significant advantages of short charging time and long cycle time (Guo et al., 2019; Kim et al., 2020c). Integrating electrochromic functions into supercapacitor energy storage devices can also realize the intelligent characteristics of visual monitoring of energy storage status while converting electrical energy (Sun et al., 2020b; Liu et al., 2021). As shown in Figure 2D, Chen et al. (Chen et al., 2014) used PANI as the cathode for stretchable electrochromic supercapacitors (ECSCs), which was able to deliver a specific capacitance of 308.4 F g^−1^, and excellent electrochemical performance is maintained even after being stretched or bent for 1,000 times. Graphene oxide (GO) and PANI can be combined into composite nanoflakes as cathode for ECSC, which exhibits excellent electrochromic and stable supercapacitor performance (Zhang et al., 2019c). Compared with PANI, GO/PANI composite material shows more agile switching speed and greater coloring efficiency. Meanwhile, it exhibits a surface capacitance of 137 mF cm^−2^, which is much higher than that of pure PANI of 36 mF cm^−2^. Furthermore, the GO/PANI-based ECSCs also show a high areal capacitance of 75 mF cm^−2^ at 0.075 mA cm^−2^, which is much higher than the 40 mF cm^−2^ of pure PANI. This significant improvement in performance is attributed to the nanostructure of the composite, which not only enlarges the reaction area for charge transfer and increases the redox reaction sites, but also facilitates the diffusion of ions and improves the utilization of active species (Zhou et al., 2016; Yun et al., 2017; Zhong et al., 2017; Kim et al., 2022). ECSCs appear to be dark blue after charging to 0.8 V, and gradually turn to be light yellow during discharge. The color change is reversible during charge/discharge. Therefore, the color of ECSCs can be also used as a judgment indicator for the storage state of capacitor capacitance.

### Electrochromic solar cells devices

In addition to monitoring the energy storage state of energy devices, electrochromic materials also have the ability to monitor the intensity of sunlight, and change color or transmittance at the same time (Ahn et al., 2007; Ling et al., 2022). This is because electrochromic devices can make visual changes with the change of applied voltage or current, and solar cells can change the output voltage of the device according to the light intensity, so the combination of thees two devices is used to control indoor temperature smart glass design (Balan et al., 2010; Qiang et al., 2013; Jena and Choudhury., 2022). As shown in Figure 2E, Ling et al. (Ling et al., 2021) combined viologens-based electrochromic device and perovskite solar cell (PSC) into a multifunctional device. A voltage is applied to the electrochromic device by the solar cell, so as to realize the multi-function of regulating the transmittance or color of the electrochromic device by altering the intensity of sunlight, and realize the dynamic regulation of the indoor temperature. When the sunlight intensity is high, the solar cell has a high output voltage, and the electrochromic smart glass shows a strong color, which can effectively isolate the penetration of sunlight and reduce the indoor temperature. When the sunlight intensity is weakened, the output voltage of the solar cell decreases, the color of the smart glass begins to fade, the sunlight can enter the room and raise the room temperature. While in the dark condition, the output voltage of the solar cell is not enough to change the color of the smart window, that is, it returns to a transparent state, which realizes the dynamic and intelligent adjustment of the room temperature (Figure 2F).

## Conclusion and outlook

In the era of rapid development of energy storage devices, integrating electrochromic multifunction into energy storage devices is a very promising design strategy. Organic materials have attracted much attention due to their advantages of obvious color difference at different states of charge, fast response speed, and easy preparation. The type and matching scheme of electrode materials and electrolytes have a great influence on the electrochemical performance of the device, as well as the stability and response speed of electrochromic. Therefore, the correct selection of materials that meet the application conditions is very important to stabilize the performance of the device. First, selecting electrode materials and electrolytes with high adaptability can not only stabilize or improve electrochemical performance, but also enable electrochromic properties with fast response, stable discoloration, and high color contrast when applied to energy storage devices. In general, most inorganic materials are better than organic materials in terms of cycle stability, so combining organic electrochromic materials and inorganic electrochromic materials to prepare composite materials, which can achieve excellent electrochromic and electrochemical performance while enriching color changes. Besides, Nano-sized materials can effectively increase the reaction area of the materials, increase the REDOX reaction sites, promote the diffusion of ions, and improve the utilization rate of active substances, thereby effectively improving the overall performance of the system. Equally importantly, we need enrich the application scope and application scenarios of organic electrochromic materials, and realize the multi-functional application of organic electrochromic materials.

## References

[B1] Ahn K.-S. Yoo S. J. Kang M.-S. Lee J.-W. Sung Y.-E. (2007). Tandem dye-sensitized solar cell-powered electrochromic devices for the photovoltaic-powered smart window. J. Power Sources 168, 533–536. 10.1016/j.jpowsour.2006.12.114

[B2] An T. Ling Y. Gong S. Zhu B. Zhao Y. Dong D. (2018). A wearable second skin‐like multifunctional supercapacitor with vertical gold nanowires and electrochromic polyaniline. Adv. Mat. Technol. 4, 1800473. 10.1002/admt.201800473

[B3] Balan A. Baran D. Sariciftci N. S. Toppare L. (2010). Electrochromic device and bulk heterojunction solar cell applications of poly 4, 7-bis(2, 3-dihydrothieno[3, 4-b] [1, 4]dioxin-5-yl)-2-dodecyl-2H-benzo[1, 2, 3]triazole (PBEBT). Sol. Energy Mat. Sol. Cells 94, 1797–1802. 10.1016/j.solmat.2010.05.048

[B4] Cai G. Chen J. Xiong J. Lee-Sie Eh A. Wang J. Higuchi M. (2020a). Molecular level assembly for high-performance flexible electrochromic energy-storage devices. ACS Energy Lett. 5, 1159–1166. 10.1021/acsenergylett.0c00245

[B5] Cai G. Cui P. Shi W. Morris S. Lou S. N. Chen J. (2020b). One-dimensional pi-d conjugated coordination polymer for electrochromic energy storage device with exceptionally high performance. Adv. Sci. 7, 1903109. 10.1002/advs.201903109 PMC7578889 33101842

[B6] Cai G. Darmawan P. Cui M. Wang J. Chen J. Magdassi S. (2016). Highly stable transparent conductive silver grid/PEDOT:PSS electrodes for integrated bifunctional flexible electrochromic supercapacitors. Adv. Energy Mat. 6, 1501882. 10.1002/aenm.201501882

[B7] Cai G. F. Zhu R. Liu S. Y. Wang J. H. Wei C. Y. Griffith K. J. (2022). Tunable intracrystal cavity in tungsten bronze-like bimetallic oxides for electrochromic energy storage. Adv. Energy Mat. 12, 2103106. 10.1002/aenm.202103106

[B8] Cai G. Wang X. Cui M. Darmawan P. Wang J. Eh A. L.-S. (2015). Electrochromo-supercapacitor based on direct growth of NiO nanoparticles. Nano Energy 12, 258–267. 10.1016/j.nanoen.2014.12.031

[B9] Chen J. Wang Z. Chen Z. Cong S. Zhao Z. (2020). Fabry-perot cavity-type electrochromic supercapacitors with exceptionally versatile color tunability. Nano Lett. 20, 1915–1922. 10.1021/acs.nanolett.9b05152 32091911

[B10] Chen X. Lin H. Chen P. Guan G. Deng J. Peng H. (2014). Smart, stretchable supercapacitors. Adv. Mat. 26, 4444–4449. 10.1002/adma.201400842 24789733

[B11] Chen Z. Mei S. Li W. Xu N. Dong Y. Jin Y. (2021). Study of multi-electron redox mechanism via electrochromic behavior in hexaazatrinaphthylene-based polymer as the cathode of lithium–organic batteries. J. Mat. Chem. A 9, 27010–27018. 10.1039/d1ta07323k

[B12] Davy N. C. Sezen-Edmonds M. Gao J. Lin X. Liu A. Yao N. (2017). Pairing of near-ultraviolet solar cells with electrochromic windows for smart management of the solar spectrum. Nat. Energy 2, 17104. 10.1038/nenergy.2017.104

[B13] Dewan A. Sur S. Narayanan R. Thotiyl M. O. (2022). MOF‐Derived carbon embedded NiO for an alkaline Zn−NiO electrochromic battery. ChemElectroChem 9. 10.1002/celc.202200001

[B14] Eh A. L.-S. Chen J. Zhou X. Ciou J.-H. Lee P. S. (2021). Robust trioptical-state electrochromic energy storage device enabled by reversible metal electrodeposition. ACS Energy Lett. 6, 4328–4335. 10.1021/acsenergylett.1c01632

[B15] Elool Dov N. Shankar S. Cohen D. Bendikov T. Rechav K. Shimon L. J. W. (2017). Electrochromic metallo-organic nanoscale films: Fabrication, color range, and devices. J. Am. Chem. Soc. 139, 11471–11481. 10.1021/jacs.7b04217 28702992

[B16] Feng J. F. Liu T. F. Cao R. (2020). An electrochromic hydrogen-bonded organic framework film. Angew. Chem. Int. Ed. 59, 22392–22396. 10.1002/anie.202006926 32885555

[B17] Guo Q. Li J. Zhang B. Nie G. Wang D. (2019). High-performance asymmetric electrochromic-supercapacitor device based on poly(indole-6-carboxylicacid)/TiO_2_ nanocomposites. ACS Appl. Mat. Interfaces 11, 6491–6501. 10.1021/acsami.8b19505 30665294

[B18] Guo W. Cong Z. Guo Z. H. Zhang P. Chen Y. Hu W. (2021). Multifunctional self‐charging electrochromic supercapacitors driven by direct‐current triboelectric nanogenerators. Adv. Funct. Mat. 31, 2104348. 10.1002/adfm.202104348

[B19] Guo Y. Li W. Yu H. Perepichka D. F. Meng H. (2017). Flexible asymmetric supercapacitors via spray coating of a new electrochromic donor-acceptor polymer. Adv. Energy Mat. 7, 1601623. 10.1002/aenm.201601623

[B20] Huang Y. Hu H. Huang Y. Zhu M. Meng W. Liu C. (2015). From industrially weavable and knittable highly conductive yarns to large wearable energy storage textiles. ACS Nano 9, 4766–4775. 10.1021/acsnano.5b00860 25842997

[B21] Huang Y. Liu J. Wang J. Hu M. Mo F. Liang G. (2018). An intrinsically self-healing NiCo||Zn rechargeable battery with a self-healable ferric-ion-crosslinking sodium polyacrylate hydrogel electrolyte. Angew. Chem. Int. Ed. 57, 9810–9813. 10.1002/anie.201805618 29905394

[B22] Huang Y. Zhu M. Huang Y. Pei Z. Li H. Wang Z. (2016). Multifunctional energy storage and conversion devices. Adv. Mat. 28, 8344–8364. 10.1002/adma.201601928 27434499

[B23] In Y. R. Kim Y. M. Lee Y. Choi W. Y. Kim S. H. Lee S. W. (2020). Ultra-low power electrochromic heat shutters through tailoring diffusion-controlled behaviors. ACS Appl. Mat. Inter. 12, 30635–30642. 10.1021/acsami.0c05918 32519836

[B24] Jang Y. J. Kim S. Y. Kim Y. M. Lee J. K. Moon H. C. (2021). Unveiling the diffusion-controlled operation mechanism of all-in-one type electrochromic supercapacitors: Overcoming slow dynamic response with ternary gel electrolytes. Energy Storage Mat. 43, 20–29. 10.1016/j.ensm.2021.08.038

[B25] Jena S. R. Choudhury J. (2022). Solar cell-coupled metallo-supramolecular polymer-based electrochromic device in renewable energy storage and on-demand usage. Sol. Energy Mat. Sol. Cells 239, 111660. 10.1016/j.solmat.2022.111660

[B26] Ji Z. Wang H. Chen Z. Wang P. Liu J. Wang J. (2020). A both microscopically and macroscopically intrinsic self-healing long lifespan yarn battery. Energy Storage Mat. 28, 334–341. 10.1016/j.ensm.2020.03.020

[B27] Jia X. Baird E. C. Blochwitz-Nimoth J. Reineke S. Vandewal K. Spoltore D. (2021). Selectively absorbing small-molecule solar cells for self-powered electrochromic windows. Nano Energy 89, 106404. 10.1016/j.nanoen.2021.106404

[B28] Kim J. Inamdar A. I. Jo Y. Cho S. Aqueel Ahmed A. T. Hou B. (2020c). Nanofilament array embedded tungsten oxide for highly efficient electrochromic supercapacitor electrodes. J. Mat. Chem. A 8, 13459–13469. 10.1039/d0ta01728k

[B29] Kim J. Rémond M. Kim D. Jang H. Kim E. (2020b). Electrochromic conjugated polymers for multifunctional smart windows with integrative functionalities. Adv. Mat. Technol. 5, 1900890. 10.1002/admt.201900890

[B30] Kim S. Y. Jang Y. J. Kim Y. M. Lee J. K. Moon H. C. (2022). Tailoring diffusion dynamics in energy storage ionic conductors for high‐performance, multi‐function, single‐layer electrochromic supercapacitors. Adv. Funct. Mat. 32, 2200757. 10.1002/adfm.202200757

[B31] Kim S. Y. Yun T. Y. Yu K. S. Moon H. C. (2020a). Reliable, high-performance electrochromic supercapacitors based on metal-doped nickel oxide. ACS Appl. Mat. Interfaces 12, 51978–51986. 10.1021/acsami.0c15424 33166118

[B32] Kim Y. Han M. Kim J. Kim E. (2018). Electrochromic capacitive windows based on all conjugated polymers for a dual function smart window. Energy Environ. Sci. 11, 2124–2133. 10.1039/c8ee00080h

[B33] Laschuk N. O. Ahmad R. Ebralidze II Poisson J. Easton E. B. Zenkina O. V. (2020). Multichromic monolayer terpyridine-based electrochromic materials. ACS Appl. Mat. Interfaces 12, 41749–41757. 10.1021/acsami.0c11478 32870639

[B34] Lee Y. Yun J. Seo M. Kim S. J. Oh J. Kang C. M. (2020). Full-color-tunable nanophotonic device using electrochromic tungsten trioxide thin film. Nano Lett. 20, 6084–6090. 10.1021/acs.nanolett.0c02097 32603122

[B35] Lei P. Y. Wang J. H. Zhang P. Liu S. Y. Zhang S. Y. Gao Y. H. (2021). Growth of a porous NiCoO_2_ nanowire network for transparent-to-brownish grey electrochromic smart windows with wide-band optical modulation. J. Mat. Chem. C 9, 14378–14387. 10.1039/d1tc03805b

[B36] Li G. Zhang B. Wang J. Zhao H. Ma W. Xu L. (2019d). Electrochromic poly(chalcogenoviologen)s as anode materials for high-performance organic radical lithium-ion batteries. Angew. Chem. Int. Ed. 58, 8468–8473. 10.1002/anie.201903152 30951238

[B37] Li H. F. Han C. P. Huang Y. Huang Y. Zhu M. S. Pei Z. X. (2018b). An extremely safe and wearable solid-state zinc ion battery based on a hierarchical structured polymer electrolyte. Energy Environ. Sci. 11, 941–951. 10.1039/c7ee03232c

[B38] Li H. Firby C. J. Elezzabi A. Y. (2019a). Rechargeable aqueous hybrid Zn^2+^/Al^3+^ electrochromic batteries. Joule 3, 2268–2278. 10.1016/j.joule.2019.06.021 30803069

[B39] Li H. McRae L. Firby C. J. Elezzabi A. Y. (2019c). Rechargeable aqueous electrochromic batteries utilizing Ti-substituted tungsten molybdenum oxide based Zn^2+^ ion intercalation cathodes. Adv. Mat. 31, e1807065. 10.1002/adma.201807065 30803069

[B40] Li J. Levitt A. Kurra N. Juan K. Noriega N. Xiao X. (2019b). MXene-conducting polymer electrochromic microsupercapacitors. Energy Storage Mat. 20, 455–461. 10.1016/j.ensm.2019.04.028

[B41] Li W. Zhang X. Chen X. Zhao Y. Wang L. Chen M. (2020). Effect of independently controllable electrolyte ion content on the performance of all-solid-state electrochromic devices. Chem. Eng. J. 398, 125628. 10.1016/j.cej.2020.125628

[B42] Li X. Du Z. Song Z. Li B. Wu L. Liu Q. (2018a). Bringing hetero-polyacid-based underwater adhesive as printable cathode coating for self-powered electrochromic aqueous batteries. Adv. Funct. Mat. 28, 1800599. 10.1002/adfm.201800599

[B43] Li X. Wang Z. Chen K. Zemlyanov D. Y. You L. Mei J. (2021). Stabilizing hybrid electrochromic devices through pairing electrochromic polymers with minimally color-changing ion-storage materials having closely matched electroactive voltage windows. ACS Appl. Mat. Interfaces 13, 5312–5318. 10.1021/acsami.0c19685 33470091

[B44] Liang G. Liu Z. Mo F. Tang Z. Li H. Wang Z. (2018). Self-healable electroluminescent devices. Light. Sci. Appl. 7, 102. 10.1038/s41377-018-0096-8 30534371 PMC6281662

[B45] Ling H. Wu J. Su F. Tian Y. Jun Liu Y. (2022). High performance electrochromic supercapacitors powered by perovskite-solar-cell for real-time light energy flow control. Chem. Eng. J. 430, 133082. 10.1016/j.cej.2021.133082

[B46] Ling H. Wu J. Su F. Tian Y. Liu Y. J. (2021). Automatic light-adjusting electrochromic device powered by perovskite solar cell. Nat. Commun. 12, 1010. 10.1038/s41467-021-21086-7 33579925 PMC7881180

[B47] Liu J. Nie N. Wang H. Chen Z. Ji Z. Duan X. (2020b). A zinc ion yarn battery with high capacity and fire retardancy based on a SiO_2_ nanoparticle doped ionogel electrolyte. Soft Matter 16, 7432–7437. 10.1039/d0sm00996b 32756666

[B48] Liu L. Diao X. He Z. Yi Y. Wang T. Wang M. (2020a). High-performance all-inorganic portable electrochromic Li-ion hybrid supercapacitors toward safe and smart energy storage. Energy Storage Mat. 33, 258–267. 10.1016/j.ensm.2020.08.023

[B49] Liu L. Du K. He Z. Wang T. Zhong X. Ma T. (2019). High-temperature adaptive and robust ultra-thin inorganic all-solid-state smart electrochromic energy storage devices. Nano Energy 62, 46–54. 10.1016/j.nanoen.2019.04.079

[B50] Liu L. Wang T. He Z. Yi Y. Wang M. Luo Z. (2021). All-solid-state electrochromic Li-ion hybrid supercapacitors for intelligent and wide-temperature energy storage. Chem. Eng. J. 414, 128892. 10.1016/j.cej.2021.128892

[B51] Liu Y. Li W. Cheng L. Liu Q. Wei J. Huang Y. (2022). Anti-freezing strategies of electrolyte and their application in electrochemical energy devices. Chem. Rec., e202200068. 10.1002/tcr.202200068 35621364

[B52] Lv H. Yang S. Li C. Han C. Tang Y. Li X. (2021). Suppressing passivation layer of Al anode in aqueous electrolytes by complexation of H_2_PO^4−^ to Al^3+^ and an electrochromic Al ion battery. Energy Storage Mat. 39, 412–418. 10.1016/j.ensm.2021.04.044

[B53] Ming S. Li Z. Zhen S. Liu P. Jiang F. Nie G. (2020). High-performance D-A-D type electrochromic polymer with π spacer applied in supercapacitor. Chem. Eng. J. 390, 124572. 10.1016/j.cej.2020.124572

[B54] Mo F. Liang G. Meng Q. Liu Z. Li H. Fan J. (2019). A flexible rechargeable aqueous zinc manganese-dioxide battery working at -20 °C. Energy Environ. Sci. 12, 706–715. 10.1039/c8ee02892c

[B55] Pei Z. Yuan Z. Wang C. Zhao S. Fei J. Wei L. (2020). A flexible rechargeable zinc-air battery with excellent low-temperature adaptability. Angew. Chem. Int. Ed. 59, 4793–4799. 10.1002/anie.201915836 31916361

[B56] Poh W. C. Gong X. Yu F. Lee P. S. (2021). Electropolymerized 1D growth coordination polymer for hybrid electrochromic aqueous zinc battery. Adv. Sci. (Weinh). 8, e2101944. 10.1002/advs.202101944 34532997 PMC8564436

[B57] Qiang P. Chen Z. Yang P. Cai X. Tan S. Liu P. (2013). TiO_2_ nanowires for potential facile integration of solar cells and electrochromic devices. Nanotechnology 24, 435403. 10.1088/0957-4484/24/43/435403 24107414

[B58] Qin S. Zhang Q. Yang X. Liu M. Sun Q. Wang Z. L. (2018). Hybrid piezo/triboelectric-driven self-charging electrochromic supercapacitor power package. Adv. Energy Mat. 8, 1800069. 10.1002/aenm.201800069

[B59] Salles P. Pinto D. Hantanasirisakul K. Maleski K. Shuck C. E. Gogotsi Y. (2019). Electrochromic effect in titanium carbide MXene thin films produced by dip‐coating. Adv. Funct. Mat. 29, 1809223. 10.1002/adfm.201809223

[B60] Sassi M. Salamone M. M. Ruffo R. Patriarca G. E. Mari C. M. Pagani G. A. (2016). State-of-the-Art neutral tint multichromophoric polymers for high-contrast see-through electrochromic devices. Adv. Funct. Mat. 26, 5240–5246. 10.1002/adfm.201601819

[B61] Shi Y. Sun M. Zhang Y. Cui J. Shu X. Wang Y. (2020). Rational design of oxygen deficiency-controlled tungsten oxide electrochromic films with an exceptional memory effect. ACS Appl. Mat. Interfaces 12, 32658–32665. 10.1021/acsami.0c06786 32610893

[B62] Stec G. J. Lauchner A. Cui Y. Nordlander P. Halas N. J. (2017). Multicolor electrochromic devices based on molecular plasmonics. ACS Nano 11, 3254–3261. 10.1021/acsnano.7b00364 28225586

[B63] Sun S. Tang C. Jiang Y. Wang D. Chang X. Lei Y. (2020a). Flexible and rechargeable electrochromic aluminium-ion battery based on tungsten oxide film electrode. Sol. Energy Mat. Sol. Cells 207, 110332. 10.1016/j.solmat.2019.110332

[B64] Sun Y. Zhu G. Zhao X. Kang W. Li M. Zhang X. (2020b). Solution-processable, hypercrosslinked polymer via post-crosslinking for electrochromic supercapacitor with outstanding electrochemical stability. Sol. Energy Mat. Sol. Cells 215, 110661. 10.1016/j.solmat.2020.110661

[B65] Tong Z. Kang T. Wan Y. Yang R. Wu Y. Shen D. (2021). A Ca‐ion electrochromic battery via a water‐in‐salt electrolyte. Adv. Funct. Mat. 31, 2104639. 10.1002/adfm.202104639

[B66] Tong Z. Lian R. Yang R. Kang T. Feng J. Shen D. (2022). An aqueous aluminum-ion electrochromic full battery with water-in-salt electrolyte for high-energy density. Energy Storage Mat. 44, 497–507. 10.1016/j.ensm.2021.11.001

[B67] Wang C. Zhang X. Liu S. Zhang H. Wang Q. Zhang C. (2021). Interfacial charge transfer and zinc ion intercalation and deintercalation dynamics in flexible multicolor electrochromic energy storage devices. ACS Appl. Energy Mat. 5, 88–97. 10.1021/acsaem.1c02508

[B68] Wang J. Liu J. Hu M. Zeng J. Mu Y. Guo Y. (2018). A flexible, electrochromic, rechargeable Zn//PPy battery with a short circuit chromatic warning function. J. Mat. Chem. A 6, 11113–11118. 10.1039/c8ta03143f

[B69] Wang Y. Jiang H. Zheng R. Pan J. Niu J. Zou X. (2020). A flexible, electrochromic, rechargeable Zn-ion battery based on actiniae-like self-doped polyaniline cathode. J. Mat. Chem. A 8, 12799–12809. 10.1039/d0ta04203j

[B70] Wang Y. Zhong X. Liu X. Lu Z. Su Y. Wang M. (2022). A fast self-charging and temperature adaptive electrochromic energy storage device. J. Mat. Chem. A 10, 3944–3952. 10.1039/d1ta10726g

[B71] Xu T. Walter E. C. Agrawal A. Bohn C. Velmurugan J. Zhu W. (2016). High-contrast and fast electrochromic switching enabled by plasmonics. Nat. Commun. 7, 10479. 10.1038/ncomms10479 26814453 PMC4737852

[B72] Yang B. Ma D. Zheng E. Wang J. (2019). A self-rechargeable electrochromic battery based on electrodeposited polypyrrole film. Sol. Energy Mat. Sol. Cells 192, 1–7. 10.1016/j.solmat.2018.12.011

[B73] Yun T. G. Kim D. Kim Y. H. Park M. Hyun S. Han S. M. (2017). Photoresponsive smart coloration electrochromic supercapacitor. Adv. Mat. 29, 1606728. 10.1002/adma.201606728 28640386

[B74] Zhai Y. Li Y. Zhang H. Yu D. Zhu Z. Sun J. (2019). Self-rechargeable-battery-driven device for simultaneous electrochromic windows, ROS biosensing, and energy storage. ACS Appl. Mat. Interfaces 11, 28072–28077. 10.1021/acsami.9b08715 31310090

[B75] Zhang K. Li N. Wang Y. Ma X. Zhao J. Qiang L. (2018). Bifunctional urchin-like WO_3_@PANI electrodes for superior electrochromic behavior and lithium-ion battery. J. Mat. Sci. Mat. Electron. 29, 14803–14812. 10.1007/s10854-018-9617-8

[B76] Zhang P. Zhu F. Wang F. Wang J. Dong R. Zhuang X. (2017). Stimulus-responsive micro-supercapacitors with ultrahigh energy density and reversible electrochromic window. Adv. Mat. 29, 1604491. 10.1002/adma.201604491 27922733

[B77] Zhang S. Cao S. Zhang T. Lee J. Y. (2020). Plasmonic oxygen-deficient TiO_2-x_ nanocrystals for dual-band electrochromic smart windows with efficient energy recycling. Adv. Mat. 32, e2004686. 10.1002/adma.202004686 32954545

[B78] Zhang S. Cao S. Zhang T. Yao Q. Lin H. Fisher A. (2019a). Overcoming the technical challenges in Al anode–based electrochromic energy storage windows. Small Methods 4, 1900545. 10.1002/smtd.201900545

[B79] Zhang S. Chen S. Cao Y. Yang F. Peng H. Yan B. (2019c). Polyaniline nanoparticle coated graphene oxide composite nanoflakes for bifunctional multicolor electrochromic and supercapacitor applications. J. Mat. Sci. Mat. Electron. 30, 13497–13508. 10.1007/s10854-019-01717-y

[B80] Zhang W. Li H. Al‐Hussein M. Elezzabi A. Y. (2019b). Electrochromic battery displays with energy retrieval functions using solution‐processable colloidal vanadium oxide nanoparticles. Adv. Opt. Mat. 8, 1901224. 10.1002/adom.201901224

[B81] Zhong Y. Chai Z. Liang Z. Sun P. Xie W. Zhao C. (2017). Electrochromic asymmetric supercapacitor windows enable direct determination of energy status by the naked eye. ACS Appl. Mat. Interfaces 9, 34085–34092. 10.1021/acsami.7b10334 28884570

[B82] Zhou F. Ren Z. Zhao Y. Shen X. Wang A. Li Y. Y. (2016). Perovskite photovoltachromic supercapacitor with all-transparent electrodes. ACS Nano 10, 5900–5908. 10.1021/acsnano.6b01202 27159013

[B83] Zhou S. Wang S. Zhou S. Xu H. Zhao J. Wang J. (2020). An electrochromic supercapacitor based on an MOF derived hierarchical-porous NiO film. Nanoscale 12, 8934–8941. 10.1039/d0nr01152e 32267275

[B84] Zhu M. S. Wang Z. G. Li H. F. Xiong Y. Liu Z. X. Tang Z. J. (2018). Light-permeable, photoluminescent microbatteries embedded in the color filter of a screen. Energy Environ. Sci. 11, 2414–2422. 10.1039/c8ee00590g

